# Low serum zinc levels and risk of incident atrial fibrillation/flutter: a multi-institutional study

**DOI:** 10.3389/fnut.2026.1746078

**Published:** 2026-02-20

**Authors:** I-Wen Chen, Li-Chen Chang, Yi-Chen Lai, Ping-Hsin Liu, Kuo-Chuan Hung

**Affiliations:** 1 Department of Anesthesiology, Chi Mei Medical Center, Liouying, Tainan city, Taiwan; 2 Department of Anesthesiology, E-Da Hospital, I-Shou University, Kaohsiung city, Taiwan; 3 Department of Anesthesiology, Chi Mei Medical Center, Tainan city, Taiwan

**Keywords:** arrhythmia, atrial fibrillation, cardiovascular risk factors, micronutrients, zinc deficiency

## Abstract

**Background:**

Atrial fibrillation/flutter (AF) is increasingly prevalent, and identification of modifiable risk factors is a priority. Zinc deficiency (ZD) has been implicated in cardiovascular disease, but large-scale evidence linking ZD to incident AF remains limited.

**Methods:**

We conducted a multi-institutional retrospective cohort study using the TriNetX Research Network, analyzing patients aged ≥40 years with documented serum zinc levels (index date) between 2010 and 2023. Patients were categorized as zinc-deficient (< 70 μg/dL, *n* = 61,732) or zinc-sufficient (70–120 μg/dL, n = 61,732) after 1:1 propensity score matching. The primary outcome was newly diagnosed AF within two years after the index date. The secondary outcomes included risks of pneumonia (positive control), ventricular fibrillation/flutter, and ischemic stroke. Outcome events were stratified into early and late onset, defined as those occurring within 1–6 months and 6–24 months, respectively.

**Results:**

ZD was associated with significantly increased AF risk during both the early (hazard ratios (HRs) 1.62, 95% confidence intervals (CIs) 1.39–1.90, *p* < 0.001) and late follow-up periods (HR 1.42, 95%CI 1.29–1.57, *p* < 0.001). A clear dose-response relationship was observed when comparing different levels of ZD to the control group. Severe ZD (< 50 μg/dL) conferred nearly two-fold higher late AF risk vs. controls (HR 2.04, 95% CI 1.67–2.49), while mild-to-moderate ZD (50–70 μg/dL) showed a modest but significant increase compared to controls (HR 1.26, 95% CI 1.14–1.41). The association between ZD and AF remained consistent across diverse patient subgroups and both pre-pandemic (2010–2019) and pandemic periods (2020–2023). The risks of pneumonia (early: HR 1.56; late: HR 1.40) and ischemic stroke (early: HR 1.19; late: HR 1.12) were also elevated in zinc-deficient patients, whereas ventricular fibrillation/flutter was not significantly associated with ZD.

**Conclusion:**

ZD may serve as an important independent risk factor for incident AF, highlighting the potential value of incorporating zinc status into cardiovascular risk assessment and considering zinc supplementation as a cost-effective preventive strategy.

## 1 Introduction

The global prevalence of atrial fibrillation/flutter (AF) is steadily rising and poses an increasing public health challenge. A nationwide study from Denmark reported a cumulative prevalence of AF of 3.0% (1), while global estimates indicated an AF prevalence of 0.51% in 2017, with projections suggesting a potential 60% increase by 2050 (2). AF not only increases the risk of stroke and heart failure but also contributes to cognitive decline, physical disability, and reduced quality of life, particularly among older adults (3, 4). Moreover, AF imposes a substantial healthcare burden, with significantly higher hospitalization rates and annual medical costs, up to $27,896 more per patient than those without AF (5, 6). Although traditional risk factors, such as hypertension, diabetes, heart failure, and aging, are well-established contributors to AF (7), they do not fully explain its growing incidence. Recent studies have identified a wider range of modifiable risk factors, including obesity, smoking, alcohol consumption, physical inactivity, and obstructive sleep apnea, all of which contribute significantly to atrial remodeling and the development of AF (7–9). As the global population age and AF prevalence continue to rise, there is an urgent need to shift attention toward AF prevention through early identification and management of potential risk factors.

Zinc, an essential trace element, functions as a cofactor for more than 300 enzymes and is vital for numerous physiological processes (10). Within the cardiovascular system, zinc has been implicated in the pathophysiology of heart failure, vascular calcification, and myocardial infarction (11). Additionally, zinc has emerged as a potential modulator of cardiac rhythm owing to its critical role in maintaining cellular homeostasis (12). Several small-scale studies have reported the potential association between zinc deficiency and development of AF. For instance, lower zinc levels have been linked to a higher incidence of postoperative AF in patients undergoing coronary artery bypass grafting (13). Similarly, research conducted on elderly patients admitted to medical intensive care units reported a significant association between low zinc concentration and the occurrence of AF (14). However, these studies provide limited evidence regarding the role of zinc deficiency in the development of new-onset AF, largely due to the small sample sizes and inherent limitations of the study design. Given the growing clinical burden of AF and the biological plausibility of zinc involvement, we conducted a large-scale investigation to evaluate whether zinc deficiency is an independent risk factor for incident AF.

## 2 Methods

### 2.1 Data source

We conducted a multi-institutional, retrospective cohort study utilizing the TriNetX Research Network, a comprehensive and privacy-compliant platform that aggregates de-identified electronic health records (EHRs) from more than 140 healthcare organizations. This real-world dataset enables access to a wide range of structured clinical information, including diagnoses, procedures, laboratory test results, medications, and outcomes, thereby supporting large-scale observational analyses. TriNetX ensures compliance with the HIPAA Privacy Rule by removing all patient identifiers, which allows for robust analysis without compromising individual privacy. This platform has been widely adopted in clinical research across disciplines such as surgery, chronic disease epidemiology, and population health (15–18). The study protocol was reviewed and approved by the Institutional Review Board of Chi Mei Medical Center (IRB number: 11310-E04), which determined that informed consent was not required due to the use of fully de-identified and retrospective data.

### 2.2 Study population

This study included individuals aged 40 years and older who had documented serum zinc levels between January 1, 2010, and December 31, 2023. Patients were categorized according to their zinc status at the time of testing: those with serum zinc concentrations below 70 μg/dL were assigned to the zinc-deficient group, whereas those with levels between 70 and 120 μg/dL were classified as having normal zinc levels. The < 70 μg/dL threshold was based on established epidemiologic standards for biochemical zinc deficiency (19), as no validated AF-specific cut-off exists. The zinc testing date was defined as the index date for subsequent outcome tracking.

To strengthen internal validity and reduce confounding, we applied a series of prespecified exclusion criteria. First, patients with any documented diagnosis of AF (ICD-10 code: I48) prior to the index date were excluded to ensure that only new-onset arrhythmia cases were captured. Second, individuals with a history of stage 4 or 5 chronic kidney disease or end-stage renal disease were excluded, given the altered zinc metabolism and elevated cardiovascular risk associated with advanced renal dysfunction (20). We further excluded patients with a documented history of heart transplantation at any time prior to the index date, as well as those who had undergone cardiac surgery within the preceding 3 months. Additionally, to avoid confounding effects of acute physiological stress, patients were excluded if they had experienced any of the following conditions within one month before the index date: acute kidney injury, sepsis, COVID-19 infection, intensive care unit (ICU) admission, or acute myocardial infarction. Finally, individuals with corrected QT intervals greater than 500 milliseconds or less than 300 milliseconds were excluded, as such abnormal QT values are associated with an increased risk of developing arrhythmias (21).

### 2.3 Data collection and propensity score matching

For each participant, baseline demographic characteristics and clinical variables were obtained based on medical records spanning 3 years prior to the index date. To improve the balance between the comparison groups and reduce the influence of confounding variables, we conducted propensity score matching in a 1:1 ratio using a greedy nearest-neighbor approach without replacement. The propensity model incorporated key demographic variables, including age, sex, race, and body mass index, along with relevant comorbid conditions and laboratory data. In recognition of the established predictors of AF, we incorporated several cardiovascular risk factors into the matching process. These included ischemic heart disease, heart failure, mitral valve disorders, chronic obstructive pulmonary disease, and vitamin D deficiency, each of which has been independently linked to increased AF risk (22–25). Pharmacological agents known to modulate AF risk, including GLP-1 receptor agonists, SGLT2 inhibitors, and steroids use, were also included (26, 27). To further minimize bias from underlying nutritional status, we also matched the patients for zinc supplementation.

### 2.4 Outcome definitions

The primary outcome was newly diagnosed AF within 2 years after the index date. To reduce misclassification and exclude undetected pre-existing cases, a 30-day washout period was applied, and events during this interval were excluded. This approach minimized the risk of capturing arrhythmias present at the time of zinc testing. The secondary outcomes included risks of ventricular fibrillation/flutter, pneumonia, and ischemic stroke. Pneumonia was included as a positive control outcome because of the established links between zinc deficiency and increased infection risk via impaired immune function (19). Outcome events were stratified into early and late onset, defined as those occurring within 1–6 months and 6–24 months, respectively.

### 2.5 Sensitivity analysis

To evaluate the robustness of our findings and exclude potential confounding factors from the COVID-19 pandemic, we conducted two sensitivity analyses that restricted the study period to different timeframes. The first analysis included patients with serum zinc level testing between January 1, 2010, and December 31, 2019, representing the pre-pandemic era when healthcare delivery patterns remained stable. The second analysis focused on the pandemic period from January 1, 2020, to December 31, 2023, capturing the period of healthcare system disruption and altered patient care patterns. This stratified approach allowed us to assess whether the observed association between zinc deficiency and AF remained consistent across different healthcare environments and potential confounding factors related to pandemic-induced changes in medical practice and patient behavior.

### 2.6 Dose-response relationship

To investigate the potential dose-response relationship and strengthen causal inference, we subdivided patients with zinc deficiency into two severity categories based on serum zinc concentrations: mild-to-moderate deficiency (50–70 μg/dL) and severe deficiency (< 50 μg/dL). We then conducted two separate comparisons against the same control group with normal zinc levels (70–120 μg/dL). The first comparison examined mild-to-moderate zinc deficiency vs. the control group, whereas the second evaluated severe zinc deficiency vs. the control group. This analytical approach was designed to determine whether there was a biological gradient in the association, with progressively higher risks corresponding to more severe degrees of zinc deficiency, thereby providing additional evidence of a potential causal relationship between zinc status and AF development.

### 2.7 Statistical analysis

Descriptive analyses were employed to characterize the baseline demographic and clinical features of the study population. Continuous data are presented as mean values with accompanying standard deviations, while categorical variables are summarized using frequencies and percentages. To assess the effectiveness of propensity score matching, we calculated standardized mean differences (SMDs); a threshold of < 0.1 was used to indicate satisfactory balance between cohorts. Additionally, propensity score distributions were visually examined to verify adequate group overlap.

For time-to-event outcomes, Kaplan–Meier curves were generated to illustrate survival probabilities, and intergroup comparisons were performed using the log-rank test. Cox proportional hazards regression was employed to estimate the risk of events over time, and results were reported as hazard ratios (HRs) with corresponding 95% confidence intervals. These models account for censoring and time-varying risks. All statistical procedures were executed using the built-in functions of the TriNetX platform, with significance defined using a two-sided *p*-value threshold of 0.05.

## 3 Results

### 3.1 Patient selection and baseline characteristics

**Figure 1:**
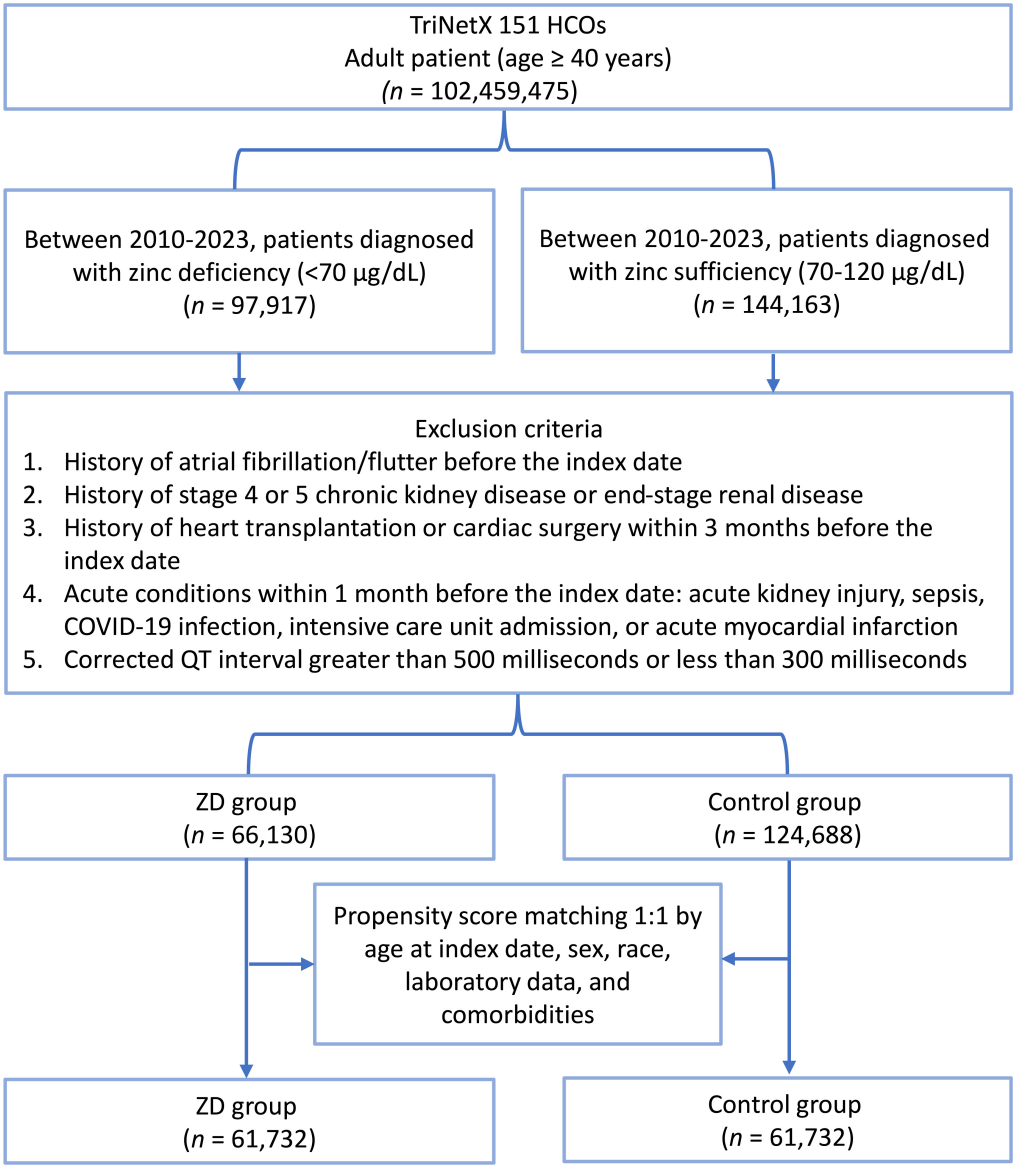
Patient selection flowchart from the TriNetX database. The flowchart illustrates the systematic exclusion process applied to identify eligible patients with zinc deficiency (ZD) and zinc sufficiency (control group). HCOs, Healthcare Organizations.

**Figure 2:**
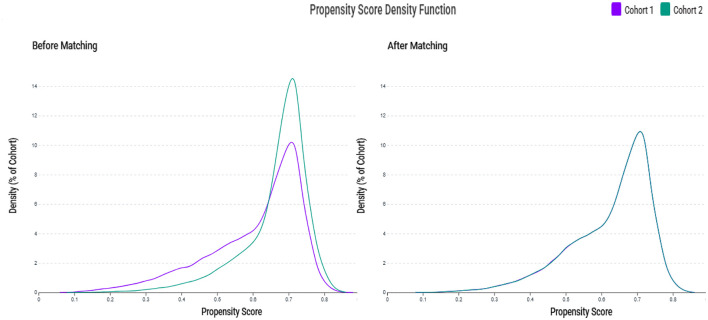
Propensity score density distributions before and after matching. The left panel shows the distribution of propensity scores between the zinc deficiency group (Cohort 1, purple) and the control group (Cohort 2, green) before matching, indicating a noticeable imbalance. The right panel shows improved overlap and covariate balance after 1:1 propensity score matching using age, sex, race, laboratory data, and comorbidities.

A total of 102,459,475 adult patients (aged ≥40 years) were identified in the TriNetX database (Figure 1). Among them, 97,917 had zinc deficiency (< 70 μg/dL) and 144,163 had zinc sufficiency (70–120 μg/dL) between 2010 and 2023. After applying the exclusion criteria, 66,130 zinc-deficient and 124,688 zinc-sufficient patients remained. Propensity score matching (1:1) based on demographics, laboratory data, and comorbidities yielded two matched cohorts of 61,732 patients each. The propensity score density plots demonstrated a notable imbalance between the zinc-deficient and control groups before matching, with distinct distributions (Figure 2). After matching, the distributions were nearly identical, indicating a successful covariate balance between the two cohorts.

Pre-matching analysis revealed substantial baseline differences, with zinc-deficient patients exhibiting higher rates of anemia (15.0% vs. 8.6%), liver disease (12.2% vs. 7.1%), nicotine dependence (8.3% vs. 5.1%), chronic obstructive pulmonary disease (4.6% vs. 2.7%), alcohol-related disorders (4.9% vs. 1.8%), and heart failure (3.6% vs. 1.8%; Table 1). The propensity score matching algorithm achieved an excellent covariate balance, with all SMDs below 0.1. The matched cohorts exhibited comparable demographic characteristics, with mean ages of 55.3 ± 13.7 and 55.1 ± 13.2 years, respectively. Cardiovascular risk factors were also well balanced, including hypertension (28.3% vs. 28.7%), diabetes mellitus (14.0% vs. 14.1%), ischemic heart disease (6.4% in both groups), and heart failure (2.9% vs. 2.8%).

**Table 1:** Baseline characteristics of patients before and after propensity score matching.

**Variables**	**Before matching**			**After matching**		
	**ZD group (*****n*** = **66,130)**	**Control group (*****n*** = **124,688)**	**SMD** ^†^	**ZD group (*****n*** = **61,732)**	**Control group (*****n*** = **61,732)**	**SMD** ^†^
**Patient characteristics**						
Age at index (years)	55.6 ± 13.8	53.9 ± 12.7	0.134	55.3 ± 13.7	55.1 ± 13.2	0.010
Female	45,044 (68.1%)	85,147 (68.3%)	0.004	42,526 (68.9%)	43,424 (70.3%)	0.032
BMI kg/m2	30.3 ± 9.4	31.0 ± 8.9	0.077	30.6 ± 9.3	30.9 ± 9.0	0.029
White	44,190 (66.8%)	86,566 (69.4%)	0.056	41,585 (67.4%)	41,190 (66.7%)	0.014
Black or African American	8,652 (13.1%)	12,156 (9.7%)	0.105	7,628 (12.4%)	7,897 (12.8%)	0.013
Asian	1,177 (1.8%)	2,381 (1.9%)	0.010	1,115 (1.8%)	1,124 (1.8%)	0.001
**Comorbidities**						
Essential (primary) hypertension	19,611 (29.7%)	32,277 (25.9%)	0.084	17,499 (28.3%)	17,726 (28.7%)	0.008
Overweight and obesity	15,756 (23.8%)	29,199 (23.4%)	0.010	14,876 (24.1%)	15,215 (24.6%)	0.013
Dyslipidemia	14,743 (22.3%)	29,981 (24.0%)	0.042	13,821 (22.4%)	14,048 (22.8%)	0.009
Neoplasms	14,773 (22.3%)	23,256 (18.7%)	0.091	13,067 (21.2%)	13,300 (21.5%)	0.009
Vitamin D deficiency	11,428 (17.3%)	20,835 (16.7%)	0.015	10,603 (17.2%)	10,788 (17.5%)	0.008
Diabetes mellitus	9,694 (14.7%)	15,467 (12.4%)	0.066	8,620 (14.0%)	8,702 (14.1%)	0.004
Anemias	9,903 (15.0%)	10,778 (8.6%)	0.197	7,799 (12.6%)	7,868 (12.7%)	0.003
Obstructive sleep apnea	7,189 (10.9%)	13,827 (11.1%)	0.007	6,828 (11.1%)	6,974 (11.3%)	0.008
Diseases of liver	8,077 (12.2%)	8,890 (7.1%)	0.173	6,237 (10.1%)	6,169 (10.0%)	0.004
Nicotine dependence	5,493 (8.3%)	6,376 (5.1%)	0.128	4,296 (7.0%)	4,318 (7.0%)	0.001
Ischemic heart diseases	4,744 (7.2%)	6,169 (4.9%)	0.093	3,930 (6.4%)	3,968 (6.4%)	0.003
COPD	3,067 (4.6%)	3,326 (2.7%)	0.105	2,385 (3.9%)	2,431 (3.9%)	0.004
CKD	2,962 (4.5%)	3,294 (2.6%)	0.099	2,372 (3.8%)	2,364 (3.8%)	0.001
COVID-19	2,216 (3.4%)	3,960 (3.2%)	0.010	2,054 (3.3%)	2,119 (3.4%)	0.006
Alcohol related disorders	3,250 (4.9%)	2,214 (1.8%)	0.175	1,978 (3.2%)	1,958 (3.2%)	0.002
Heart failure	2,381 (3.6%)	2,234 (1.8%)	0.112	1,763 (2.9%)	1,753 (2.8%)	0.001
Long term use of steroids	1,984 (3.0%)	2,379 (1.9%)	0.071	1,647 (2.7%)	1,618 (2.6%)	0.003
Other venous embolism and thrombosis	2,049 (3.1%)	2,023 (1.6%)	0.097	1,489 (2.4%)	1,539 (2.5%)	0.005
Nonrheumatic mitral valve disorders	1,236 (1.9%)	1,801 (1.4%)	0.033	1,037 (1.7%)	1,053 (1.7%)	0.002
Rheumatic mitral valve diseases	149 (0.2%)	199 (0.2%)	0.015	126 (0.2%)	125 (0.2%)	< 0.001
**Laboratory data**						
Hemoglobin ≥ 12 mg/dL	39,633 (59.9%)	77,349 (62.0%)	0.043	37,340 (60.5%)	37,822 (61.3%)	0.016
Hemoglobin A1c < 7%	25,033 (37.9%)	48,444 (38.9%)	0.021	23,423 (37.9%)	23,574 (38.2%)	0.005
Albumin g/dL (≥3.5 g/dL)	37,996 (57.5%)	70,059 (56.2%)	0.026	35,358 (57.3%)	36,541 (59.2%)	0.039
eGFR > 60 mL/min/1.73 m^2^	45,219 (68.4%)	79,311 (63.6%)	0.101	41,229 (66.8%)	41,581 (67.4%)	0.012
**Medications**						
Antiarrhythmics	21,647 (32.7%)	30,581 (24.5%)	0.182	18,678 (30.3%)	19,104 (30.9%)	0.015
Beta blockers/related	13,226 (20.0%)	19,661 (15.8%)	0.111	11,449 (18.5%)	11,627 (18.8%)	0.007
Antilipemic agents	11,663 (17.6%)	21,185 (17.0%)	0.017	10,653 (17.3%)	10,804 (17.5%)	0.006
Calcium channel blockers	7,572 (11.5%)	11,556 (9.3%)	0.072	6,635 (10.7%)	6,706 (10.9%)	0.004
Insulin	7,653 (11.6%)	9,253 (7.4%)	0.142	6,256 (10.1%)	6,391 (10.4%)	0.007
Antianginals	3,198 (4.8%)	4,412 (3.5%)	0.065	2,700 (4.4%)	2,780 (4.5%)	0.006
GLP-1 analogs	2,150 (3.3%)	4,104 (3.3%)	0.002	2,077 (3.4%)	2,129 (3.4%)	0.005
SGLT2 inhibitors	739 (1.1%)	1,496 (1.2%)	0.008	697 (1.1%)	745 (1.2%)	0.007
Zinc supplements	1,864 (2.8%)	2,820 (2.3%)	0.035	1,603 (2.6%)	1,666 (2.7%)	0.006
Antihypertensive combinations	739 (1.1%)	1,496 (1.2%)	0.008	697 (1.1%)	745 (1.2%)	0.007

ZD, zinc deficiency; BMI, body mass index; SMD, standardized mean differences;^†^SMD values < 0.1 indicate adequate balance between groups; GLP-1, glucagon-like peptide-1; SGLT2, sodium-glucose co-transporter 2; CKD, chronic kidney disease; COPD, chronic obstructive pulmonary disease; eGFR, estimated Glomerular filtration rate.

### 3.2 Association between zinc deficiency and 2-year AF risk

Our primary analysis revealed a significant association between zinc deficiency and incident AF, demonstrating both immediate and sustained cardiovascular risks (Table 2). During the early stage (1–6 months post-index), zinc-deficient patients exhibited a 62% higher risk than controls (HR 1.62, 95% CI 1.39–1.90; *p* < 0.001). This elevated risk pattern persisted throughout the extended late-stage follow-up period (6–24 months), maintaining a 42% increased hazard for AF development (HR 1.42, 95% CI 1.29–1.57; *p* < 0.001).

**Table 2:** Association Between zinc deficiency and risk of atrial fibrillation/flutter.

**Outcomes**	**ZD group (*n* = 61,732)**	**Control group (*n* = 61,732)**	**HR (95% CI)**	***p*-value**
	**Events (%)**	**Events (%)**		
**Early stage (1–6 month)**				
AF	392 (0.64%)	249 (0.40%)	1.62 (1.39–1.90)	< 0.001
Pneumonia	1,152 (1.9%)	764 (1.2%)	1.56 (1.42–1.70)	< 0.001
Ventricular fibrillation/Flutter	14 (0.02%)	14 (0.02%)	1.03 (0.49–2.16)	0.939
Ischemic stroke	729 (1.2%)	631 (1.0%)	1.19 (1.07–1.32)	0.002
**Late stage (6–24 month)**				
AF	891 (1.4%)	664 (1.1%)	1.42 (1.29–1.57)	< 0.001
Pneumonia	2,074 (3.4%)	1,575 (2.6%)	1.40 (1.31–1.49)	< 0.001
Ventricular fibrillation/Flutter	33 (0.05%)	23 (0.04%)	1.52 (0.89–2.58)	0.123
Ischemic stroke	1,145 (1.9%)	1,080 (1.7%)	1.12 (1.03–1.22)	0.008

ZD, zinc deficiency; HR, hazard ratio; CI, confidence interval; AF, atrial fibrillation/flutter.

Examination of secondary outcomes provided biological validation of our primary findings. Pneumonia incidence, strategically selected as a positive control due to zinc's well-established immunomodulatory functions, was significantly higher in zinc-deficient patients during both the early (HR 1.56, 95% CI 1.42–1.70) and late stages (HR 1.40, 95% CI 1.31–1.49). This pattern strongly supports the biological plausibility of our cardiovascular findings, as it aligns with the critical roles of zinc in immune function and cellular metabolism.

Interestingly, ventricular fibrillation/flutter risk showed no significant difference between the groups, suggesting that zinc deficiency specifically influences atrial rather than ventricular arrhythmogenesis. Despite the increased AF risk, ischemic stroke elevation was relatively mild (early: HR, 1.19; late: HR, 1.12), suggesting that the observed increase in AF did not translate into proportionally higher thromboembolic complications.

### 3.3 Sensitivity analysis on AF risk at late stage across different study periods

To evaluate whether the observed associations reflected true biological relationships rather than artifacts of healthcare practices, we analyzed the effects of zinc deficiency across two distinct timeframes (Table 3). During the pre-pandemic period (2010–2019, *n* = 31,638 matched pairs), zinc deficiency was strongly associated with an increased AF risk (HR 1.49, 95% CI 1.29–1.73; *p* < 0.001). This association persisted, although attenuated, during the pandemic era (2020–2023, *n* = 35,702 pairs; HR 1.34, 95% CI 1.17–1.53; *p* < 0.001). A similar pattern was observed for pneumonia (HR 1.58 vs. 1.30 pre-pandemic), consistent with increased susceptibility to respiratory infections. In contrast, ventricular fibrillation/flutter remained non-significant in both periods. These findings reinforce the biological plausibility of zinc deficiency as a risk factor for AF, independent of external healthcare system fluctuations.

**Table 3:** Sensitivity analysis: Association between zinc deficiency and atrial fibrillation/flutter at late stage (6–24 month after index date).

**Outcomes**	**2010–2019** ^**†**^		**2020–2023** ^**‡**^	
	**HR (95% CI)**	***p*** **-values**	**HR (95% CI)**	***p*** **-values**
AF	1.49 (1.29–1.73)	< 0.001	1.34 (1.17–1.53)	< 0.001
Pneumonia	1.30 (1.18–1.42)	< 0.001	1.58 (1.45–1.72)	< 0.001
Ventricular fibrillation/flutter	0.98 (0.50–1.94)	0.959	0.85 (0.44–1.64)	0.627
Ischemic stroke	1.05 (0.93–1.19)	0.436	1.20 (1.08–1.34)	< 0.001

^†^*n* = 31,638 for each group; ^‡^*n* = 35,702 for each group; ZD, zinc deficiency; HR, hazard ratio; CI, confidence interval; AF, atrial fibrillation/flutter.

### 3.4 Dose-response relationship

Our analysis demonstrated a clear biological gradient, reinforcing causality (Table 4). Patients with mild-to-moderate zinc deficiency (50–70 μg/dL; *n* = 56,206 per group) exhibited modest but statistically significant risks of AF during both early (HR 1.40, 95% CI 1.18–1.67) and late follow-up (HR 1.26, 95% CI 1.14–1.41). In contrast, individuals with severe zinc deficiency (< 50 μg/dL; *n* = 8,961 per group) showed markedly elevated risks, with a nearly threefold increase in early follow-up (HR 2.79) and a twofold increase in late follow-up (HR 2.04). This strong dose-response relationship meets Bradford Hill's criterion for causality (28), indicating that even mild deficiency poses a cardiovascular risk, whereas severe deficiency significantly amplifies that risk. Similar trends were observed for pneumonia, with severe deficiency associated with elevated hazards in both the early (HR 2.27) and late (HR 1.92) phases. However, while ischemic stroke risk remained elevated in mild-to-moderate deficiency, the association was not seen in the severely deficient subgroup, likely due to reduced statistical power from the smaller sample size.

**Table 4:** Dose-response relationship across different levels of zinc deficiency at early (1–6 m) and late-stage (6–24 m) follow up.

**Outcomes**	**Early stage**		**Late stage**	
	**HR (95% CI)**	***p*** **-values**	**HR (95% CI)**	***p*** **-values**
**Mild-to-moderate Zinc deficiency (50–70** μ**g/dL) vs. control group**^†^				
AF	1.40 (1.18–1.67)	< 0.001	1.26 (1.14–1.41)	< 0.001
Pneumonia	1.36 (1.23–1.50)	< 0.001	1.29 (1.21–1.39)	< 0.001
Ventricular fibrillation/flutter	1.11 (0.93–1.32)	0.249	1.25 (0.73–2.12)	0.413
Ischemic stroke	1.32 (1.18–1.48)	< 0.001	1.15 (1.05–1.25)	0.002
**Severe Zinc level (**<**50**μ**g/dL) vs. control group**^‡^				
AF	2.79 (2.08–3.74)	< 0.001	2.04 (1.67–2.49)	< 0.001
Pneumonia	2.27 (1.94–2.66)	< 0.001	1.92 (1.69–2.17)	< 0.001
Ventricular fibrillation/flutter	1.30 (0.44–3.87)	0.636	0.67 (0.25–1.82)	0.431
Ischemic stroke	1.15 (0.90–1.46)	0.256	1.11 (0.91–1.35)	0.290

^†^*n* = 56,206 for each group; ^‡^*n* = 8,961 for each group; HR: hazard ratio; CI, confidence interval; AF, atrial fibrillation/flutter.

### 3.5 Subgroup analysis

Subgroup analysis demonstrated remarkable consistency across diverse patient populations, underscoring the generalizability of our findings (Table 5). The zinc deficiency-AF association remained significant in all examined strata, with risks ranging from 1.26 to 1.61. Both men (HR 1.33) and women (HR 1.50) showed significant associations, although women demonstrated a numerically higher risk. Consistency across traditional cardiovascular risk factors is particularly noteworthy. For example, patients with or without hypertension, diabetes, heart failure, or ischemic heart disease all demonstrated significant associations, suggesting that zinc deficiency contributes additional risk beyond these conventional conditions. No significant interaction was observed across sex, age, or comorbidity (all *p* > 0.05), indicating that the adverse effects of zinc deficiency are broadly applicable.

**Table 5:** Subgroup analyses of association between zinc deficiency and risk of atrial fibrillation/flutter at late-stage.

**Subgroup analysis**	**Number of each group**	**HR (% CI)**	***p*-value**	***p* for interaction**
**Sex**				
Male	16,293	1.33 (1.15–1.54)	<0.001	Reference
Female	42,928	1.50 (1.30–1.73)	<0.001	0.251
**Age**				
40–65 years	39,095	1.53 (1.25–1.87)	<0.001	Reference
>65 years	22,446	1.35 (1.20–1.51)	<0.001	0.309
**Dyslipidemia**				
Yes	18,156	1.38 (1.20–1.60)	<0.001	Reference
No	43,542	1.39 (1.21–1.59)	<0.001	0.943
**Hypertension**				
Yes	21,623	1.31 (1.16–1.49)	<0.001	Reference
No	39,998	1.61 (1.38–1.89)	<0.001	0.053
**Diabetes mellitus**				
Yes	10,857	1.26 (1.05–1.52)	0.014	Reference
No	50,825	1.43 (1.27–1.61)	<0.001	0.251
**Obesity**				
Yes	19,209	1.48 (1.23–1.78)	<0.001	Reference
No	42,209	1.46 (1.30–1.65)	<0.001	0.904
**Heart failure**				
Yes	2,531	1.40 (1.07–1.82)	0.014	Reference
No	59,099	1.47 (1.32–1.64)	<0.001	0.737
**Ischemic heart disease**				
Yes	5,717	1.36 (1.13–1.64)	0.001	Reference
No	55,902	1.48 (1.31–1.66)	<0.001	0.447

HR, hazard ratio; CI, confidence interval.

## 4 Discussion

This large-scale, multi-institutional study provides evidence that zinc deficiency is a significant and independent risk factor for developing AF. Our analysis of over 123,000 matched patients demonstrated that individuals with serum zinc levels below 70 μg/dL exhibited a 62% increased risk of incident AF during the early follow-up period and a sustained 42% elevated risk throughout the extended observation phase. A clear dose-response relationship was observed when comparing different levels of ZD to the control group. Severe ZD conferred nearly two-fold higher late AF risk vs. controls (HR 2.04), while mild-to-moderate ZD showed a modest but significant increase compared to controls (HR 1.26). These associations remained robust across diverse patient populations and different healthcare environments, including the pre-pandemic and pandemic periods, suggesting that the relationship reflects genuine biological mechanisms rather than confounding factors related to healthcare delivery patterns.

The link between zinc deficiency and AF may be explained by multiple interconnected biological pathways that influence both cardiac electrical activity and structural remodeling. Zinc serves as a cofactor for over 300 enzymes involved in cellular metabolism, protein synthesis, and antioxidant defense systems. In cardiac tissues, zinc deficiency disrupts calcium homeostasis by impairing calcium-handling proteins, leading to altered excitation-contraction coupling and increased susceptibility to triggered arrhythmias (29, 30). This mineral also plays a crucial role in maintaining cellular membrane stability through its involvement in phospholipid metabolism and membrane-bound enzyme function (31, 32). Furthermore, zinc deficiency promotes oxidative stress by reducing the activity of zinc-dependent antioxidant enzymes such as superoxide dismutase (31, 33). This oxidative environment facilitates atrial remodeling through fibroblast activation, collagen deposition, and inflammatory cascade activation (34). The resulting structural changes create a substrate for reentrant arrhythmias by altering the conduction velocity and refractoriness patterns. Additionally, zinc deficiency affects ion channel function, particularly potassium and sodium channels, which are critical for maintaining normal cardiac rhythm (12, 35), thereby creating an electrophysiological milieu conducive to arrhythmia initiation and maintenance.

Previous meta-analyses reported inconsistent associations between serum zinc and cardiovascular outcomes (36, 37). Chu et al. (36) found largely null associations overall, with inverse serum zinc relationships limited to high-risk subgroups, while Hashemian et al. (37) reported supportive evidence mainly from case–control studies, with inconsistent results across prospective cohorts. Subsequent studies focused on advanced cardiovascular disease and mortality-related endpoints rather than arrhythmia incidence (38, 39). Consequently, prior literature often included patients with structural heart disease or prevalent AF, limiting inference on zinc status and incident arrhythmia risk. In contrast, the present study uniquely examined incident AF as the primary outcome, using strict exclusion of pre-existing AF, comprehensive propensity score matching, and dose–response modeling. These methodological features enhance temporal inference compared with prior cross-sectional or mortality-based studies (36, 37). Clinically, our findings suggest that zinc deficiency may represent a modifiable risk factor for atrial arrhythmogenesis, and serum zinc assessment could help identify individuals who may benefit from targeted nutritional evaluation or supplementation trials.

This study is the first large-scale investigation to establish zinc deficiency as an independent risk factor for incident AF. A key strength of our analysis lies in the assessment of new-onset AF within a clearly defined follow-up window, enabling time-sequenced evaluation and enhancing the validity of causal inference. We found that zinc deficiency was associated with a significantly increased risk of developing AF, with HRs of 1.62 for early-stage and 1.42 for late-stage onset. Our findings not only extend the clinical relevance of zinc beyond heart failure and myocardial infarction (40–42), but also underscore the importance of incorporating micronutrient evaluation into cardiovascular risk stratification. The temporal pattern of the risk revealed important clinical insights. The higher early stage risk suggests an acute vulnerability period where zinc deficiency may trigger arrhythmia through immediate metabolic disruption, while sustained late-stage elevation may indicate chronic structural remodeling effects. This dual-phase pattern has not been previously described for micronutrient deficiencies in the development of arrhythmia.

Interestingly, while AF risk increased substantially, the corresponding ischemic stroke elevation was mild (12–19%). This disparity may be explained by the temporal relationship between new-onset AF and subsequent thromboembolic events. The development of ischemic stroke following incident AF typically requires time for atrial thrombus formation and embolization to occur, particularly in patients who may not have received immediate anticoagulation therapy. Given our relatively short follow-up period of 2 years, this timeframe may be insufficient to capture the full stroke risk associated with zinc deficiency-induced AF. The mild stroke elevation observed in our study likely reflects the early phase of this pathophysiological process, where the arrhythmogenic effects of zinc deficiency are immediately apparent, but the downstream thromboembolic consequences have not yet been fully manifested. This temporal delay underscores the importance of early recognition and management of zinc deficiency as a modifiable risk factor, potentially preventing both arrhythmia development and subsequent stroke complications with long-term follow-up.

Because our study period encompassed the COVID-19 pandemic, and zinc homeostasis has been implicated in COVID-19 susceptibility and disease severity (43, 44), we specifically addressed this potential confounding by performing stratified sensitivity analyses across pre-pandemic (2010–2019) and pandemic (2020–2023) periods. The association between zinc deficiency and incident AF remained consistent in both periods, indicating that our findings were unlikely to be driven by pandemic-related healthcare disruptions or infection-related biases. These results support a stable biological relationship between zinc deficiency and atrial arrhythmogenesis independent of COVID-19–related effects.

Regarding the clinical implications of our findings, healthcare providers may consider incorporating serum zinc assessment into cardiovascular risk evaluation, particularly for patients with established AF risk factors or unexplained palpitations. The dose-response relationship observed in our study suggests that even mild zinc deficiency warrants attention, as it still conferred a 26% increased risk of late-stage AF. From a therapeutic perspective, zinc supplementation represents a potentially cost-effective intervention for AF prevention. However, the optimal dosing, duration, and target population for zinc supplementation require careful investigation in randomized controlled trials. Clinicians should also be aware that zinc deficiency often coexists with other nutritional deficiencies and comorbid conditions, suggesting that comprehensive nutritional assessment and management are necessary.

The inclusion of pneumonia risk as a positive control outcome provided crucial biological validation of our primary findings. Pneumonia risk showed substantial elevation in zinc-deficient patients during both the early (HR 1.56) and late stages (HR 1.40), closely paralleling the AF pattern. This observation strongly supports the biological plausibility of our cardiovascular findings, as the role of zinc in immune function is well established (45). The established effectiveness of zinc even as a direct anti-infective, as in treatment of conditions such as cutaneous leishmaniasis (46), provides further credence to the validity of its association with infection-related outcomes, such as pneumonia, in our cohort. Parallel patterns between pneumonia and AF risks further establish our confidence that observed associations reflect real biologic relationships.

The absence of increased ventricular fibrillation/flutter risk in zinc-deficient patients provides important insights into the specificity of the arrhythmogenic effects of zinc. This finding suggests that zinc deficiency preferentially affects atrial electrophysiology rather than ventricular electrophysiology. This specificity has clinical relevance, as it indicates that zinc deficiency may represent a targeted risk factor for supraventricular rather than life-threatening ventricular arrhythmias. This observation also supports the biological plausibility of our findings by demonstrating that the association is not simply due to increased overall arrhythmia surveillance or detection bias in zinc-deficient patients. Instead, it points to specific pathophysiological mechanisms that primarily affect the atrial substrate and conduction properties.

Several limitations must be acknowledged when interpreting our findings. First, despite extensive adjustment and demonstration of a dose–response relationship, this observational study cannot establish causality. Residual confounding from unmeasured lifestyle or nutritional factors may persist. Important factors such as overall dietary quality, intake of other micronutrients (e.g., magnesium or potassium), socioeconomic status, physical activity, alcohol consumption patterns, smoking intensity, and underlying chronic inflammatory states were not fully captured and may influence both zinc status and AF risk. Second, the TriNetX database may not capture all relevant confounding variables, particularly subclinical conditions that could influence both zinc status and arrhythmia risk. For example, reverse causality remains possible, whereby subclinical or undiagnosed AF, or shared underlying conditions such as systemic inflammation or heart failure, could contribute to reduced zinc levels rather than zinc deficiency preceding AF development. Third, the definition of zinc deficiency may not represent the optimal cut-off point for cardiovascular risk stratification. Our dose–response analyses across graded deficiency levels demonstrated progressively increasing AF risk, suggesting that cardiovascular risk varies continuously with zinc status and is not dependent on a single threshold. Future prospective studies are required to define the optimal zinc cut-off for AF risk stratification. Fourth, serum zinc levels are influenced by acute-phase responses, infection, circadian variation, and recent dietary intake. Therefore, reliance on a single zinc measurement may not accurately reflect an individual's long-term zinc status or tissue-level deficiency and may result in exposure misclassification (e.g., transient reductions or unrecognized chronic deficiency). Although such non-differential misclassification would be expected to bias the observed associations toward the null, it remains an important limitation of the present study. Fifth, our analysis focused on diagnosed AF episodes, potentially missing asymptomatic or undetected cases, which could lead to underestimation of the true association. Finally, selection bias is also possible because study participants were drawn from individuals who received medical evaluation and serum zinc testing, who may differ systematically from healthier community populations. Furthermore, confounding by indication cannot be excluded, as the clinical rationale for zinc testing was unavailable and may have been influenced by suspected malnutrition or higher baseline cardiovascular risk. Accordingly, our findings indicate association rather than causation, and prospective interventional studies are required to confirm any preventive effect of zinc supplementation on AF.

## 5 Conclusion

Our analysis of over 123,000 patients demonstrates an association between zinc deficiency and increased risk of incident AF, with a clear dose–response pattern across deficiency severity. These findings support the potential clinical value of serum zinc assessment in AF risk evaluation. However, causality cannot be established, and randomized controlled trials are required to determine whether zinc supplementation can effectively prevent AF and define optimal treatment strategies.

## Data Availability

The raw data supporting the conclusions of this article will be made available by the authors, without undue reservation.

## References

[1] Hegelund ER Kjerpeseth LJ Mortensen LH Igland J Berge T Anjum M . Prevalence and incidence rates of atrial fibrillation in Denmark 2004–2018. Clin Epidemiol. (2022) 14:1193–204. doi: 10.2147/CLEP.S374468 36325198 PMC9618383

[2] Lippi G Sanchis-Gomar F Cervellin G. Global epidemiology of atrial fibrillation: an increasing epidemic and public health challenge. Int J Stroke. (2021) 16:217–21. doi: 10.1177/1747493019897870 31955707

[3] Bencivenga L Komici K Nocella P Grieco FV Spezzano A Puzone B . Atrial fibrillation in the elderly: a risk factor beyond stroke. Ageing Res Rev. (2020) 61:101092. doi: 10.1016/j.arr.2020.101092 32479927

[4] Sanoski CA. Clinical, economic, and quality of life impact of atrial fibrillation. J Manag Care Pharm. (2009) 15:S4–9. doi: 10.18553/jmcp.2009.15.s6-b.4 19678721 PMC10442900

[5] Deshmukh A Iglesias M Khanna R Beaulieu T. Healthcare utilization and costs associated with a diagnosis of incident atrial fibrillation. Heart Rhythm O2. (2022) 3:577–86. doi: 10.1016/j.hroo.2022.07.010 36340482 PMC9626881

[6] Weir MR Chen YW He J Bookhart B Campbell A Ashton V. Healthcare resource utilization and costs of rivaroxaban versus warfarin among nonvalvular atrial fibrillation patients with obesity and diabetes. Diabetes Ther. (2021) 12:3167–86. doi: 10.1007/s13300-021-01161-4 34699020 PMC8586051

[7] Bizhanov KA. Abzaliyev KB, Baimbetov AK, Sarsenbayeva AB, Lyan E. Atrial fibrillation: epidemiology, pathophysiology, and clinical complications (literature review). J Cardiovasc Electrophysiol. (2023) 34:153–65. doi: 10.1111/jce.15759 36434795

[8] Shamloo AS Dagres N Arya A Hindricks G. Atrial fibrillation: a review of modifiable risk factors and preventive strategies. Rom J Intern Med. (2019) 57:99–109. doi: 10.2478/rjim-2018-0045 30648669

[9] Du X Dong J Ma C. Is atrial fibrillation a preventable disease? J Am Coll Cardiol. (2017) 69:1968–82. doi: 10.1016/j.jacc.2017.02.020 28408027

[10] Cheng Y Chen H. Aberrance of zinc metalloenzymes-induced human diseases and its potential mechanisms. Nutrients. (2021) 13:124456. doi: 10.3390/nu13124456 34960004 PMC8707169

[11] Begum F Me HM Christov M. The role of zinc in cardiovascular disease. Cardiol Rev. (2022) 30:100–8. doi: 10.1097/CRD.0000000000000382 35119422

[12] Kokhabi P Mollazadeh R Hejazi SF Nezhad AH Pazoki-Toroudi H. Importance of zinc homeostasis for normal cardiac rhythm. Curr Cardiol Rev. (2025) 21:e1573403X299868. doi: 10.2174/011573403X299868240904120621 39301907 PMC12060914

[13] Yan YQ Zou LJ. Relation between zinc, copper, and magnesium concentrations following cardiopulmonary bypass and postoperative atrial fibrillation in patients undergoing coronary artery bypass grafting. Biol Trace Elem Res. (2012) 148:148–53. doi: 10.1007/s12011-012-9356-2 22351155

[14] Aly K Shaat M Hamza S Ali S. Triggers of atrial fibrillation in the geriatric medical intensive care unit: an observational study. Cardiol Res. (2023) 14:106–14. doi: 10.14740/cr1461 37091882 PMC10116932

[15] Wu JY Chen CC Ling Tu W Hsu WH Liu TH Tsai YW . Clinical impact of tirzepatide on patients with OSA and obesity. Chest. (2025) 168:785–96. doi: 10.1016/j.chest.2025.03.030 40254150

[16] Ho CN Chung WC Kao CL Hsu CW Hung KC Yu CH . Impact of preoperative QTc interval prolongation on short-term postoperative outcomes: a retrospective study. J Clin Anesth. (2024) 98:111574. doi: 10.1016/j.jclinane.2024.111574 39121785

[17] Chen IW Chang LC Ho CN Wu JY Tsai YW Lin CM . Association between COVID-19 and the development of chronic kidney disease in patients without initial acute kidney injury. Sci Rep. (2025) 15:10924. doi: 10.1038/s41598-025-96032-4 40158028 PMC11954888

[18] Ho CN Sun CK Wu JY Chen JY Chang YJ Chen IW . Association of vitamin D deficiency with post-stroke depression: a retrospective cohort study from the TriNetX US collaborative networks. Front Nutr. (2023) 10:1236233. doi: 10.3389/fnut.2023.1236233 37599698 PMC10436528

[19] Meydani SN Barnett JB Dallal GE Fine BC Jacques PF Leka LS . Serum zinc and pneumonia in nursing home elderly. Am J Clin Nutr. (2007) 86:1167–73. doi: 10.1093/ajcn/86.4.1167 17921398 PMC2323679

[20] Elgenidy A Amin MA Awad AK Husain-Syed F Aly MG. Serum zinc levels in chronic kidney disease patients, hemodialysis patients, and healthy controls: systematic review and meta-analysis. J Ren Nutr. (2023) 33:103–15. doi: 10.1053/j.jrn.2022.04.004 35472507

[21] Tse G Chan YW Keung W Yan BP. Electrophysiological mechanisms of long and short QT syndromes. Int J Cardiol Heart Vasc. (2017) 14:8–13. doi: 10.1016/j.ijcha.2016.11.006 28382321 PMC5368285

[22] Liu X Wang W Tan Z Zhu X Liu M Wan R . The relationship between vitamin D and risk of atrial fibrillation: a dose-response analysis of observational studies. Nutr J. (2019) 18:73. doi: 10.1186/s12937-019-0485-8 31727055 PMC6857145

[23] Huang Q Xiong H Shuai T Zhang M Zhang C Wang Y . Risk factors for new-onset atrial fibrillation in patients with chronic obstructive pulmonary disease: a systematic review and meta-analysis. PeerJ. (2020) 8:e10376. doi: 10.7717/peerj.10376 33344074 PMC7718784

[24] Goudis CA. Chronic obstructive pulmonary disease and atrial fibrillation: An unknown relationship. J Cardiol. (2017) 69:699–705. doi: 10.1016/j.jjcc.2016.12.013 28188041

[25] Benjamin EJ Levy D Vaziri SM D'Agostino RB Belanger AJ Wolf PA. Independent risk factors for atrial fibrillation in a population-based cohort. The Framingham Heart Study. JAMA. (1994) 271:840–4. doi: 10.1001/jama.1994.03510350050036 8114238

[26] Chan YH Chao TF Chen SW Lee HF Li PR Chen WM . The risk of incident atrial fibrillation in patients with type 2 diabetes treated with sodium glucose cotransporter-2 inhibitors, glucagon-like peptide-1 receptor agonists, and dipeptidyl peptidase-4 inhibitors: a nationwide cohort study. Cardiovasc Diabetol. (2022) 21:118. doi: 10.1186/s12933-022-01549-x 35765074 PMC9241240

[27] Christiansen CF Christensen S Mehnert F Cummings SR Chapurlat RD Sørensen HT. Glucocorticoid use and risk of atrial fibrillation or flutter: a population-based, case-control study. Arch Intern Med. (2009) 169:1677–83. doi: 10.1001/archinternmed.2009.297 19822824

[28] Davidson TM Smith WM. The Bradford Hill criteria and zinc-induced anosmia: a causality analysis. Arch Otolaryngol Head Neck Surg. (2010) 136:673–6. doi: 10.1001/archoto.2010.111 20644061

[29] Dorward AM Stewart AJ Pitt SJ. The role of Zn2+ in shaping intracellular Ca^2+^ dynamics in the heart. J Gen Physiol. (2023) 155:13206. doi: 10.1085/jgp.202213206 37326614 PMC10276528

[30] Lee SR Noh SJ Pronto JR Jeong YJ Kim HK Song IS . The critical roles of zinc: beyond impact on myocardial signaling. Korean J Physiol Pharmacol. (2015) 19:389–99. doi: 10.4196/kjpp.2015.19.5.389 26330751 PMC4553398

[31] Marreiro DD Cruz KJ Morais JB Beserra JB Severo JS de Oliveira AR. Zinc and oxidative stress: current mechanisms. Antioxidants. (2017) 6: 24. doi: 10.3390/antiox6020024 28353636 PMC5488004

[32] Gimenez MS Oliveros LB Gomez NN. Nutritional deficiencies and phospholipid metabolism. Int J Mol Sci. (2011) 12:2408–33. doi: 10.3390/ijms12042408 21731449 PMC3127125

[33] Choi S Liu X Pan Z. Zinc deficiency and cellular oxidative stress: prognostic implications in cardiovascular diseases. Acta Pharmacol Sin. (2018) 39:1120–32. doi: 10.1038/aps.2018.25 29926844 PMC6289396

[34] Gutierrez A Van Wagoner DR. Oxidant and inflammatory mechanisms and targeted therapy in atrial fibrillation: an update. J Cardiovasc Pharmacol. (2015) 66:523–9. doi: 10.1097/FJC.0000000000000313 26335221 PMC4674316

[35] Noh S Lee SR Jeong YJ Ko KS Rhee BD Kim N . The direct modulatory activity of zinc toward ion channels. Integr Med Res. (2015) 4:142–6. doi: 10.1016/j.imr.2015.07.004 28664120 PMC5481804

[36] Chu A Foster M Samman S. Zinc status and risk of cardiovascular diseases and type 2 diabetes mellitus—a systematic review of prospective cohort studies. Nutrients. (2016) 8:707. doi: 10.3390/nu8110707 27827959 PMC5133094

[37] Hashemian M Poustchi H Mohammadi-Nasrabadi F Hekmatdoost A. Systematic review of zinc biochemical indicators and risk of coronary heart disease. ARYA Atheroscler. (2015) 11:357–65. 26862344 PMC4738046

[38] Lin YM Tu WL Hung KC Yu T Wu JY Lai CC. Mortality and cardiorenal outcomes among heart failure patients with zinc deficiency: a multicenter retrospective cohort study of 8,290 patients. Front Nutr. (2025) 12:1589907. doi: 10.3389/fnut.2025.1589907 40357034 PMC12066520

[39] Soinio M Marniemi J Laakso M Pyörälä K Lehto S Rönnemaa T. Serum zinc level and coronary heart disease events in patients with type 2 diabetes. Diabetes Care. (2007) 30:523–8. doi: 10.2337/dc06-1682 17327315

[40] Yu X Huang L Zhao J Wang Z Yao W Wu X . The relationship between serum zinc level and heart failure: a meta-analysis. Biomed Res Int. (2018) 2018:2739014. doi: 10.1155/2018/2739014 29682528 PMC5845493

[41] Liu R Yao J Chen K Peng W. Association between biomarkers of zinc and copper status and heart failure: a meta-analysis. ESC Heart Fail. (2024) 11:2546–56. doi: 10.1002/ehf2.14837 38690587 PMC11424300

[42] Banik S Ghosh A. Zinc status and coronary artery disease: A systematic review and meta-analysis. J Trace Elem Med Biol. (2022) 73:127018. doi: 10.1016/j.jtemb.2022.127018 35709561

[43] Wang F Zhong J Zhang R Sun Y Dong Y Wang M . Zinc and COVID-19: immunity, susceptibility, severity and intervention. Crit Rev Food Sci Nutr. (2024) 64:1969–87. doi: 10.1080/10408398.2022.2119932 36094452

[44] Wessels I Rolles B Slusarenko AJ Rink L. Zinc deficiency as a possible risk factor for increased susceptibility and severe progression of Corona Virus Disease 19. Br J Nutr. (2022) 127:214–32. doi: 10.1017/S0007114521000738 33641685 PMC8047403

[45] Shankar AH Prasad AS. Zinc and immune function: the biological basis of altered resistance to infection. Am J Clin Nutr. (1998) 68:447s−63s. doi: 10.1093/ajcn/68.2.447S 9701160

[46] Firooz A Khatami A Khamesipour A Nassiri-Kashani M Behnia F Nilforoushzadeh M . Intralesional injection of 2% zinc sulfate solution in the treatment of acute old world cutaneous leishmaniasis: a randomized, double-blind, controlled clinical trial. J Drugs Dermatol. (2005) 4:73–9. 15696988

